# P54 A rare, treatable and reversible cause of fixed flexion deformity in juvenile idiopathic arthritis, not to be missed!

**DOI:** 10.1093/rap/rkae117.085

**Published:** 2024-11-05

**Authors:** Sana Sharrack, India Soar, Lisa Dunkley

**Affiliations:** Sheffield Teaching Hospitals NHS Foundation Trust, Sheffield, United Kingdom; Sheffield Teaching Hospitals NHS Foundation Trust, Sheffield, United Kingdom; Sheffield Teaching Hospitals NHS Foundation Trust, Sheffield, United Kingdom

## Abstract

**Introduction:**

Vitamin C deficiency, otherwise known as scurvy, is typically thought of as a disease of the past. However, it does still exist today in certain patient cohorts at risk of nutritional deficiencies or poor gastrointestinal absorption. We present the rare case of a 24-year-old male with known enthesitis-related juvenile idiopathic arthritis, who after years of quiescent disease on a combination of sulfasalazine and adalimumab, presented to clinic with new bilateral knee fixed flexion deformities and difficulty mobilising. After extensive investigations, extremely low vitamin C levels were confirmed and high dose supplementation led to significant improvement in knee flexion and mobility.

**Case description:**

Our patient is a 24-year-old male, born prematurely with delayed puberty, who was diagnosed with enthesitis-related juvenile idiopathic arthritis (JIA) at the age of 16 having likely had joint disease for at least two years beforehand and quite advanced disease at presentation. He responded well to sulfasalazine and adalimumab. At the time of diagnosis, he presented with a spontaneous left-sided iliopsoas bleed and thorough haematological investigations were undertaken at the time with no clear cause identified. It was at this point however that vitamin C deficiency was considered, confirmed and treated with oral ascorbic acid. Our patient was then stable for several years till he represented to clinic having sustained a fall leading to fracture and dislocation of the left elbow requiring internal fixation. Post-operatively, his left elbow became acutely inflamed and fixed with no evidence of infection (retrospectively, likely a bleed). One month later, our patient developed spontaneous bleeding into the posteromedial aspect of the left thigh associated with knee fixed flexion (and absence of knee inflammation), with similar developments in the contralateral side two weeks later. He had skin bruising, but no other bleeding. At this point, the patient was admitted for further investigations. Investigations revealed a normocytic anaemia, normal platelet count and an extensive and essentially normal coagulation screen. Haematology input was sought and the only other possible causes were vascular/true connective tissue disease or scurvy (our patient was still taking vitamin C supplementation). Extremely low vitamin C levels were confirmed despite oral replacement, thought to be due to a combination of ongoing nutritional deficiency (element of neglect), concern about compliance, and concern about absorption with evidence of stercoral colitis secondary to constipation. On subsequent follow-up, he was found to have significant improvement in bleeding, knee flexion and mobility with high dose supplementation, laxatives and intensive community dietetic input.

**Discussion:**

JIA often presents atypically compared to adult inflammatory arthritis, with cool non-swollen joints not being uncommon despite active disease. The result can be silent disease leading to restricted joint movement/mobility which can be irreversible. It is therefore often difficult at times to identify active joint disease in JIA patients and one can therefore be forgiven in assuming a fixed flexion deformity in a JIA patient is due to silent but active inflammatory joint disease. However, it is important always to look at the bigger picture and consider alternative causes for symptoms. In our patient, the skin bleeding and corkscrew hairs in the context of a patient who was clearly nutritionally deficient with a low BMI and concern about possible neglect (which prompted a safeguarding assessment) raised the possibility of something else going on. On further questioning by the haematologist, it was identified that our patient would often have only one or two meals a day and these meals almost always consisted of cereal only for both breakfast and dinner. He had not had any fruit or vegetables for several years. He also revealed to us that he was severely constipated. An inpatient CT of the abdomen and pelvis revealed severe constipation with possible stercoral colitis. This raised the possibility of poor absorption. The patient was adamant that he was taking his ascorbic acid religiously, and this may have been correct, but poor compliance was also considered as a potential cause. Through this case, we have learned about the importance of assessing patients holistically, always seeking to find out more about their living situation, dietary habits, and seek out any behaviours that raise concern for abuse, neglect or safeguarding. This is especially the case for young and vulnerable JIA patients.

**Key learning points:**

• This case has highlighted to us that scurvy is not just a disease of the past, but indeed is sadly still a disease of today, especially in certain cohorts of patients such as those with neurodevelopmental abnormalities, psychiatric illness, unusual dietary habits or who are vulnerable, abused or neglected. It is therefore vital to look at patients holistically and to involve dieticians early if there is concern about nutritional deficiency. It is easy to assume a musculoskeletal problem is due to a previously identified underlying rheumatological condition, especially when disease can be quiet. However, this case has highlighted the importance and need to consider rarer but easily treatable other causes. The case has also illustrated the importance of raising awareness of scurvy as a cause for intramuscular haematoma, causing fixed flexion deformity in the JIA cohort of patients amongst the rheumatology family and beyond. Sometimes, scurvy may be incorrectly diagnosed as JIA and patients may not receive the treatment they need to easily reverse their symptoms and prevent irreversible contractures and joint damage (amongst other more serious complications of the disease). They may also be unnecessarily exposed to dangerous immunosuppressive treatments. Finally, this case has highlighted the importance of taking a full and thorough history and performing a full and thorough examination (which helped identify skin bruising, bleeding and corkscrew hairs!). We found liaising with our haematology and genetic colleagues extremely helpful and they helped lead us to the diagnose and to rule out other causes (including vascular EDS). Help from allied health professionals including dieticians, physiotherapists and occupational therapists cannot be overstated and these professionals played a pivotal role in helping our patient regain mobility and functionality. It would be interesting to discuss at the conference whether formal dietician input should form part of routine JIA assessment.

